# A Speedy Cardiovascular Diseases Classifier Using Multiple Criteria Decision Analysis

**DOI:** 10.3390/s150101312

**Published:** 2015-01-12

**Authors:** Wah Ching Lee, Faan Hei Hung, Kim Fung Tsang, Hoi Ching Tung, Wing Hong Lau, Veselin Rakocevic, Loi Lei Lai

**Affiliations:** 1 Department of Electronic and Information Engineering, Hong Kong Polytechnic University, Hong Kong, China; E-Mail: enwclee@polyu.edu.hk; 2 Department of Electronic Engineering, City University of Hong Kong, Hong Kong, China; E-Mails: fhhung4@cityu.edu.hk (F.H.H.); itwhlau@cityu.edu.hk (W.H.L.); 3 School of Engineering & Mathematical Science, City University London, Northampton Square, London EC1V 0HB, UK; E-Mails: cherry.tung.1@city.ac.uk (H.C.T.); V.Rakocevic@city.ac.uk (V.R.); 4 Energy Strategy, Planning, Policy Support, R&D Centre, State Grid Energy Research Institute, SGCC Administrative Area, Future Science and Technology Park, Changping, Beijing 102209, China; E-Mail: lailaili@sgeri.sgcc.com.cn

**Keywords:** cardiovascular diseases classifier, electrocardiogram, multiple criteria decision analysis, analytic hierarchy process, support vector machine

## Abstract

Each year, some 30 percent of global deaths are caused by cardiovascular diseases. This figure is worsening due to both the increasing elderly population and severe shortages of medical personnel. The development of a cardiovascular diseases classifier (CDC) for auto-diagnosis will help address solve the problem. Former CDCs did not achieve quick evaluation of cardiovascular diseases. In this letter, a new CDC to achieve speedy detection is investigated. This investigation incorporates the analytic hierarchy process (AHP)-based multiple criteria decision analysis (MCDA) to develop feature vectors using a Support Vector Machine. The MCDA facilitates the efficient assignment of appropriate weightings to potential patients, thus scaling down the number of features. Since the new CDC will only adopt the most meaningful features for discrimination between healthy persons *versus* cardiovascular disease patients, a speedy detection of cardiovascular diseases has been successfully implemented.

## 1. Introduction

Electrocardiogram (ECG) signals, characterized by P waves, Q waves, S waves, QRS complexes and T waves, are important information for cardiovascular disease diagnosis by cardiologists. Such a diagnosis requires the development of a cardiovascular diseases classifier (CDC). Generally, a CDC mainly comprises feature vectors extraction and building a classifier via machine learning algorithms like an Artificial Neural Network or Support Vector Machine. Features can be divided into three categories: non-fiducial features, fiducial features, and hybrid features. Non-fiducial features normally refer to features that do not characterize the ECG signals using P waves, Q waves, S waves, QRS complexes and T waves [1–5], and *vice versa* for fiducial features [6,7]. Hybrid features refer to feature vectors constructed by both non-fiducial and fiducial features [8–10].

In this investigation, a Support Vector Machine (SVM) is utilized to construct the CDC for the four most common types of cardiovascular diseases, namely bundle branch block, myocardial infarction, heart failure, and dysrhythmia. Seven criteria, including overall accuracy (OA), sensitivity (S_e_), specificity (S_p_), area under the curve (AUC), training time (T_r_), testing time (T_e_), and number of features (N_f_), which are features indicative of the speed and accuracy of detection, are used as the essential parameters to compute the analytic hierarchy process (AHP) score to aid the multiple criteria decision analysis (MCDA) for the evaluation of the optimal CDC. Traditional work usually aims at the highest overall accuracy and/or lowest testing time. In reality, every end user has to specify the weights between criteria. It is not uncommon to find a ratio setting by intuition or simply a direct 1:1 assignment is adopted. It is noted that the practical needs of volunteers are neglected or not targeted. In the new method, assignments of criteria are devised for AHP analysis. The incorporation of AHP analysis in the classifier enables the consideration of the need of volunteers. This letter is organized as follows: the design of an optimal CDC is presented in Section 2. Multiple criteria decision analysis of the optimal CDC is given in Section 3. In Section 4, the AHP is formulated and a performance score is obtained from which the performance is analyzed and compared to traditional schemes. Finally, conclusions are drawn in Section 5.

## 2. Design of the optimal CDC

**Figure 1.**
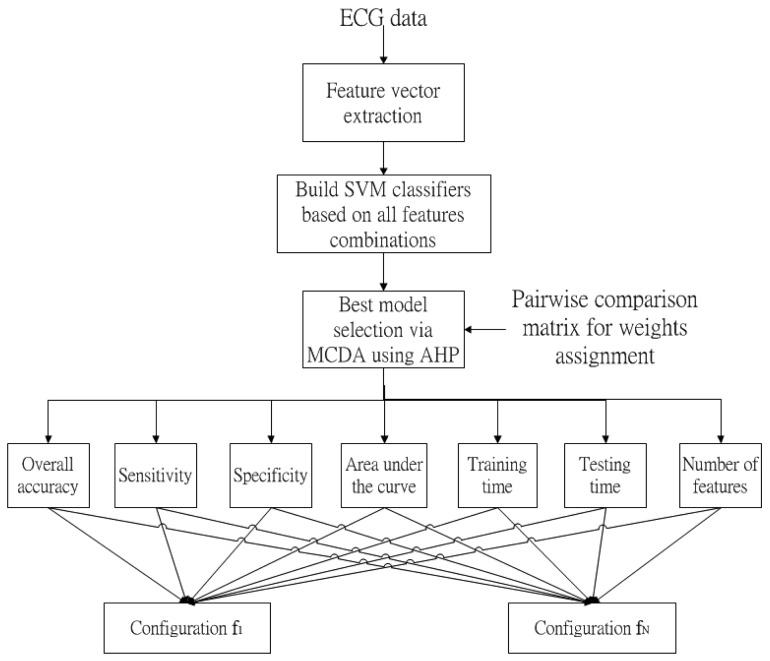
Block diagram of the new method.

Figure 1 summarizes the block diagram of the new method. After the retrieval of ECG data, feature vectors are extracted. The SVM classifiers are then designed based on the features combinations. Therefore, N configurations can be obtained. The best model is selected among configuration f_1_ to configuration f_N_ based on seven criteria, namely overall accuracy, sensitivity, specificity, area under the curve, training time, testing time, and number of features, with the aid of MCDA *via* AHP. The details of the new method are illustrated in the following.

### 2.1. Data Preprocessing and Features Construction

The data is obtained from an online and open access database [11,12]. A group of healthy candidates as well as candidates with the four most common types of cardiovascular diseases are selected: 52 candidates from health control, 15 bundle branch block candidates, 148 myocardial infarction candidates, 18 heart failure candidates and 14 dysrhythmia candidates. The unequal sample size in each class will lead to a bias of the SVM classifier [13]. The Lead I ECG signal is further partitioned into 30 s sub-signals to obtain 500 samples of healthy candidates and 125 samples of unhealthy candidates (of each type of cardiovascular disease). This process aims at equalizing the number of samples in each class (healthy and unhealthy). Before the introduction of these four diseases, the notations are briefed. Denote RR-interval to be the consecutive R points between consecutive ECG signals, QRS complex is the time between Q wave and S wave where point R is between Q wave and S wave. Similarly, QT interval refers to the time between point Q wave and T wave. The background of these four diseases is presented as follows:

(i) Myocardial Infarction: Irregular heartbeat and thus irregular RR-interval may occur in the ECG signal of the patients [14];
(ii) Bundle Branch Block: Patients have QRS complex with value exceeding 0.12 ms [15];
(iii) Dysrhythmia: The heartbeat can be more than 100 beats per minute or less than 60 beats per minute. Thus, RR-interval is different from the normal ECG signal. Also, the QT interval may increase if the type of cardiovascular disease is ventricular arrhythmias [16];
(iv) Heart Failure: A finding of prolonged QT interval in the ECG signals of the patients [17].

As a result, Q wave, R wave and S wave, QRS complex, and RR-interval are representative features to identify between healthy persons *versus* cardiovascular patients. The feature vector consists of 10 features using the average and standard deviation of these five parameters. Before detecting and computing the features, the ECG signals will undergo data preprocessing [18]. The maximum frequency of an ECG signal is typically less than 60 Hz, thus a bandpass filter with cutoff frequencies at 1 Hz and 60 Hz is implemented. A derivative filter is then applied to sharpen the Q, R, and S wave. Finally, signal squaring and sliding window integration are utilized for the location of Q, R, and S wave.

### 2.2. Cardiovascular Diseases Classifier Construction

The CDC is constructed by employing SVM with a 10-dimensional feature vector. This algorithm uses a Lagrange Multiplier with a set of support vectors, a set of weighting and an offset bias [19,20]. This report focuses on the design of CDC.

The performance of CDC is dictated by OA, S_e_, S_p_, AUC, T_r_, T_e_, and N_f_. It directly classifies the ECG signal into healthy (negative response) candidates and unhealthy (positive response) candidates. OA, S_e_, S_p_, and AUC are related to the accuracy of CDC. T_r_ is the time required to train the CDC and T_e_ is the time needed to detect the ECG signal. In this investigation, CDC will be trained up and validated with the ECG datasets. For the analysis of positive response—Class 0, 500 healthy patients are used. For the analysis of positive response—Class 1, 125 bundle branch block patients, 125 myocardial infarction patients, 125 heart failure patients and 125 dysrhthmia patients are retrieved from the database. Table 1 lists the datasets for CDC with binary classifier.

The CDC utilizes a 10-fold cross validation for performance evaluation [21] and the polynomial kernel function (third order) is utilized for SVM analysis. There is a total of 1023 combinations ( ∑n=110C10n), thus 1023 configurations can be formulated from a selection (from 1 to 10) of the 10 features. For the j^th^ configuration where j = 1,…,1023, namely f_j_, its corresponding criteria, OA, S_e_, S_p_, AUC, T_r_, T_e_, and N_f_ are recorded. The main settings of SVM are summarized as follows, in general, the default setting is utilized in the MATLAB toolbox:

(i) Number of classes: Two;
(ii) Class 0: 500 Healthy candidates;
  Class 1: 125 bundle branch block candidates, 125 myocardial infarction candidates, 125 heart failure candidates, and 125 dysrhthmia candidates;
(iii) Feature vector: The maximum dimensionality is 10, which consists of: {Q wave average, Q wave standard deviation, R wave average, R wave standard deviation, S wave average, S wave standard deviation, QRS complex average, QRS complex standard deviation, RR-interval mean, RR-interval standard deviation};
(iv) Kernel function: 3rd order polynomial;
(v) Fold of cross validation: Ten-fold 1023 classifiers are constructed in 1023 configurations; the results are tabulated in Table 2.

## 3. Multiple Criteria Decision Analysis of the Optimal CDC

In Table 2, seven criteria, namely OA, S_e_, S_p_, AUC, T_r_, T_e_, and N_f_, are employed for performance evaluation of the 1023 scenarios. Multiple criteria decision making (MCDM) has been utilized in many areas since the 1990s [22]. It entails using the particular characteristics of cardiovascular diseases. By allocating appropriate weightings, the analytic hierarchy process (AHP) is adopted to evaluate and analyze the best scenarios among the 1023 scenarios investigated. The allocation of weightings confronts the feedback from an AHP analysis of 200 volunteers from which a pairwise comparison 7 × 7 matrix A_m_ (m = 1, …, 200) is formulated. It is intuitively understood that T_e_ should be as low as possible and that the accuracy should be kept to an acceptable level. Since the speed of detection is the prime factor of importance, the analysis on MCDA reveals that high weightings should be assigned to OA, S_e_, S_p_, AUC, T_e_. These five parameters are referred as primary parameters. While N_f_ is typically preferred to be small for speedy detection, it is noted that T_r_ will not affect the detection time. Hence N_f_ and T_r_ are classified as the secondary parameters.

The volunteers are required to fill in the a_m,ij_ , where i and j are between 1 and 7, in Table 3. The AHP based MCDA CDC is referred as the new classifier (NC). Traditional classifiers (TC) in [3,7,8] are also evaluated. Both the NC and the TC are applied to the three feature groups (non-fiducially features, fiducially features and hybrid features in [3,7,8]. The performance comparison between the NC and the TC is tabulated in Table 4. Based on the discussion for AHP formulation, the assignment of values of a_m,ij_ are based on the following guidelines:

(i) Write 1 if equal importance of i and j;
(ii) Write 3 if i is slightly more important than j;
(iii) Write 5 if i is more important than j;
(iv) Write 7 if i is strongly more important than j;
(iv) Write 9 if i is absolutely more important than j.

The pairwise comparison 7 × 7 matrix A_m_ is then normalized, and Anorm_m_ can be obtained by modifying the matrix entries a_m,ij_ in A_m_ into matrix entries anorm_m,ij_ in Anorm_m_:

(1) $${\text{anorm}}_{m,ij}=\frac{a_{m,ij}}{\overset{7}{\underset{l=1}{\text{∑}}}a_{m,lj}}$$

By averaging each row of Equation (1), the corresponding 7 × 1 priority matrix w_m_ with entries w_m,k_ for k = 1,…,7 is given by:

(2) $$w_{m,k}=\frac{1}{7}\sum_{l=1}^{7}{\text{anorm}}_{m,kl}$$

Denote C_p,q_, (*p* = 1,…,7 and q = 1,…,1023) be the p^th^ criteria, and q^th^ scenario of CDC. C_p,q_ is normalized to become C_p,q,norm_. The final score for each scenario, AHP_q_, is evaluated by:

(3) $${\text{AHP}}_{q}=\sum_{l=1}^{7}C_{p,q,\text{norm}}(\frac{1}{200}\sum_{m=1}^{200}w_{m,l})$$

To avoid inconsistency in the construction of pairwise comparison matrices, the optimal CDC is concluded from the highest value of AHP_q_ [23]. It is evaluated that the optimal CDC is obtained from scenario f_652_, with feature vector composes of average of Q, standard deviation of Q, standard deviation of S, average of QRS mean, standard deviation of QRS, average of RR-interval, and standard deviation of RR-interval, with AHP_652_ as follows: OA = 0.988, S_e_ = 0.992, S_p_ = 0.985, AUC = 0.982, T_r_ = 4.5 s, T_e_ = 2.8 s, N_f_ = 7.

## 4. AHP Scores and Analysis

The performance scores between the NC and the TC [3,7,8] are evaluated and tabulated in Table 4. In this investigation, the algorithms in related work have been evaluated, with the addition of MCDA using AHP to obtain a best scenario by assigning weights to the seven criteria. As the new work and related works are in the same application area, the classification of cardiovascular diseases, the weight assignment can be reused to facilitate performance comparisons. From Table 4, the percentage changes are evaluated as follows:

1. Percentage change compared with AHP scores from [3]: OA = −0.504%, S_e_ = −0.302%, S_p_ = −0.807%, AUC = −0.515%, T_r_ = −8.109%, T_e_ = −29.630%, and N_f_ = −33.333%. It is concluded that there is an improvement of 30% in speed of detection of cardiovascular diseases @∼99.5% accuracy.
2. Percentage change compared with AHP scores from [7]: OA = −1.025%, S_e_ = 0.514%, S_p_ = −3.036%, AUC = −1.686%, T_r_ = −9.677%, T_e_ = −31.707%, and N_f_ = −33.333%. It is concluded that there is an improvement of 30% in speed of detection of cardiovascular diseases @∼99% accuracy.
3. Percentage change compared with AHP scores from [8]: OA = −0.525%, S_e_ = 0.105%, S_p_ = −0.946%, AUC = −0.636%, T_r_ = −6.250%, T_e_ = −40.741%, and N_f_ = −23.077%. It is concluded that there is an improvement of 40% in speed of detection of cardiovascular diseases @∼99.5% accuracy.

The analysis reveals that in the NC, the speed of detection has been increased by 30%–40% while the accuracy is retained at ∼99%–99.5% of the TC. It is seen that there the reduction of OA, S_e_, and S_p_ are less than 1%. Thus the AHP based MCDA CDC is a reliable and speedy detection scheme for cardiovascular diseases.

## 5. Conclusions

In this letter, an optimal cardiovascular diseases classifier (CDC) has been proposed and implemented by using an analytic hierarchy process (AHP) to facilitate multiple criteria decision analysis (MCDA). The four most common types of cardiovascular diseases, namely bundle branch block, myocardial infarction, heart failure, and dysrhythmia are considered. Seven criteria, namely OA, S_e_, S_p_, AUC, T_r_, T_e_, and N_f_ are carefully considered and chosen to be the criteria for deriving the AHP score of MCDA to achieve the optimal CDC. The optimal CDC, the new classifier, achieves the following scores: OA = 0.988, S_e_ = 0.992, S_p_ = 0.985, AUC = 0.982, T_r_ = 4.5 s, T_e_ = 2.8 s, N_f_ = 7. Analysis and comparison with previous works show that the speed of detection cardiovascular diseases has been increased by 30%–40% while the accuracy is retained at ∼99%–99.5% of traditional classifiers. In conclusion, the AHP based MCDA CDC is a reliable and speedy detection scheme for cardiovascular diseases.

## Figures and Tables

**Table 1.** Database specification of ECG data for CDC.

**Class 0 (Healthy/Negative Response)**	**Number of Samples**	**Class 1 (Unhealthy/Positive Response)**	**Number of Samples**
PTB diagnostic (Healthy)	500	Bundle Branch Block	125
		Myocardial Infarction	125
		Heart Failure	125
		Dysrhthmia	125

**Table 2.** CDC of each configuration.

**f_j_**	**OA**	**S_e_**	**S_p_**	**AUC**	**T_r_ (s)**	**T_e_ (s)**	**N_f_**
f_1_	0.324	0.350	0.298	0.321	3.5	2.3	1
f_2_	0.310	0.324	0.296	0.303	3.4	2.5	1
f_3_	0.298	0.288	0.308	0.287	3.6	2.4	1
…	…	…	…	…	…	…	…
f_1021_	0.986	0.988	0.984	0.972	4.9	3.4	10
f_1022_	0.964	0.970	0.958	0.946	5.1	3.4	10
f_1023_	0.970	0.974	0.966	0.949	4.3	3.5	10

**Table 3.** Pairwise comparison 7 × 7 matrix A_m_.

	**OA**	**S_e_**	**S_p_**	**AUC**	**T_r_**	**T_e_**	**N_f_**
OA	1	a_m,12_	a_m,13_	a_m,14_	a_m,15_	a_m,16_	a_m,17_
S_e_	a_m,21_	1	a_m,23_	a_m,24_	a_m,25_	a_m,26_	a_m,27_
S_p_	a_m,31_	a_m,32_	1	a_m,34_	a_m,35_	a_m,36_	a_m,37_
AUC	a_m,41_	a_m,42_	a_m,43_	1	a_m,45_	a_m,46_	a_m,47_
T_r_	a_m,51_	a_m,52_	a_m,53_	a_m,54_	1	a_m,56_	a_m,57_
T_e_	a_m,61_	a_m,62_	a_m,63_	a_m,64_	a_m,65_	1	a_m,67_
N_f_	a_m,71_	a_m,72_	a_m,73_	a_m,74_	a_m,75_	a_m,76_	1

**Table 4.** Performance of NC *versus* TC.

**Method**	**Datasets (Number of Samples)**	**Features**	**Results (Related Work TC)**	**Results (New Work NC)**
Two-layered Hidden Markov Model [3]	MIT-BIH database (34,799 samples from 16 Arrhythmia candidates)	P-R interval, QRS complex interval and T sub-wave interval	OA = 0.992	OA = 0.987
			S_e_ = 0.993	S_e_ = 0.99
			S_p_ = 0.992	S_p_ = 0.984
			AUC = 0.971	AUC = 0.966
			T_r_ = 3.7 s	T_r_ = 3.4 s
			T_e_ = 2.7 s	T_e_ = 1.9 s
			N_f_ = 3	N_f_ = 2

Cross wavelet transform with a threshold based classifier [7]	The PTB Diagnostic ECG database (18,489 samples from 52 healthy control candidates and 148 myocardial infarction candidates)	Total sum of wavelet cross spectrum value and total sum of wavelet coherence	OA = 0.976	OA = 0.966
			S_e_ = 0.973	S_e_ = 0.978
			S_p_ = 0.988	S_p_ = 0.958
			AUC = 0.949	AUC = 0.933
			T_r_ = 6.2 s	T_r_ = 5.6 s
			T_e_ = 4.1 s	T_e_ = 2.8 s
			N_f_ = 6	N_f_ = 4

SVM [8]	CU database, VF database, and AHA database (40,956 samples from 67 Ventricular fibrillation and rapid ventricular tachycardia candidates)	Leakage, count 1, count 2, count 3, A1, A2, A3, time delay, FSMN, cover bin, frequency bin, kurtosis, and complexity	OA = 0.952	OA = 0.947
			S_e_ = 0.951	S_e_ = 0.952
			S_p_ = 0.951	S_p_ = 0.942
			AUC = 0.943	AUC = 0.937
			T_r_ = 4.8 s	T_r_ = 4.5 s
			T_e_ = 2.7 s	T_e_ = 1.6 s
			N_f_ = 13	N_f_ = 10
